# Increasing trend of childhood type 1 diabetes incidence: 20-year observation from Greater Poland Province, Poland

**DOI:** 10.1007/s00592-024-02339-5

**Published:** 2024-07-18

**Authors:** Elżbieta Niechciał, Michał Michalak, Bogda Skowrońska, Piotr Fichna

**Affiliations:** 1 Department of Pediatric Diabetes, Clinical Auxology and Obesity, Poznan University of Medical Sciences, 27/33 Szpitalna St., Poznan, 60-572 Poland; 2 Department of Informatics and Statistics, Poznan University of Medical Sciences, 7 Rokietnicka St., Poznan, 60-529 Poland

**Keywords:** Children, Adolescents, Epidemiology, Incidence rate, Type 1 diabetes

## Abstract

**Aim:**

Type 1 diabetes is one of the fastest-growing chronic health conditions. Estimating the incidence rate of childhood type 1 diabetes will allow to aid in adequate planning of health care resources. The study’s aim was to assess the incidence rate of type 1 diabetes in children below 15 years of age from Greater Poland (Poland) between 2006 and 2018, and then to compare obtained data to records collected between 1998 and 2003 in pediatric population aged 0–14 years from the same area.

**Methods:**

In this cohort study covering the period from January 1998 to December 2018, data were collected for children and adolescents below 14 years of age with newly diagnosed type 1 diabetes living in Greater Poland. The overall population size was taken from the Statistical Office of Poland. Total, sex-, and age-specific incidence rates per 100,000 person-years were calculated for each calendar year.

**Results:**

Over a 20-year period, the incidence rate of type 1 diabetes in children aged 0–14 years rose around 3.6-fold, from 8.4/100,000 in 1998 to 30.8/100,000 in 2018, with the peak incidence recorded in last year of the study. A clear male predominance of type 1 diabetes was seen in all ages. The rate of type 1 diabetes incidence growth was comparable between all age groups, while the highest incidence rate was mostly observed in children aged 5–9 and 10–14 years.

**Conclusions:**

The incidence of type 1 diabetes in children aged 0–14 years is rapidly increasing in Greater Poland.

## Introduction

Type 1 diabetes (T1D) is one of the fastest-growing chronic health conditions [1–3]. Its acute and chronic complications pose significant challenges to the health care system [4]. The global incidence rate (IR) of T1D has been rising since the 1950s, with an average annual increase of 3–4% over the past three decades [5–7]. In 2022, the number of people living with T1D was estimated to be approximately 8.75 million worldwide, with 530,000 new cases that year. Of this group, 1.52 million (17%) were aged younger than 20 years, 5.56 million (64%) were aged between 20 and 59 years, and 1.67 million (19%) were aged 60 and older. The number of people having T1D is expected to increase rapidly and reach 17.4 million by the year 2040 [2, 8]. More detailed data on childhood T1D are regularly provided by the International Diabetes Federation (IDF). According to the most recent edition of IDF Diabetes Atlas, globally over 1.2 million children and adolescents under 20 years were estimated to have T1D in 2021, with 150,000 new cases diagnosed that year. **Nearly 54% of all existing cases of T1D are children under 15 years of age** [3]. The results of different epidemiological studies worldwide showed that the IR of T1D varies significantly in different regions and ethnic populations [9]. Europe has the greatest number of children having T1D, with the most rapid rate of increase contributed by countries in Central and Eastern Europe. Caucasians people seem to be more susceptible to T1D than other ethnic groups. The highest IR is still seen in children younger than 15 years in Finland and Sweden (52.2 and 44.1/100,000 per year, respectively). However, upward trends are reported from a number of non-Europid populations. For example, Kuwait now has the third highest IR in the world (41.1/100,000 per year) followed by Qatar, Canada or Algeria (38.1, 37.9 and 34.8/100,000 per year, respectively) [3, 9, 10]. In the United States, the IR T1D in children and adolescents is estimated around 22.3/100,000 per year, with significant differences between race/ethnic groups (27.3/100,000 per year in non-Hispanic White youth, 20.8/100,000 per year in Black youth, and 16.3/100,000 per year in Hispanic youth) [11]. In contrast, the lowest IR is seen across East and South-East Asia [3]. Nevertheless, estimating the global epidemiology of T1D is challenging as data arises from country-specific studies conducted using different methodologies. In many countries, there are no national registries, while in other countries completeness of registries is uncertain as case ascertainments are often under-estimated. The IR a key element in tracking the progress of epidemics of chronic disease such as T1D in order to determine both current and future health care resources required to maintain the health of this population who are at significant risk of life-threating complications.

The main purpose of this study was to assess the IR of T1D in children below 15 years of age from Greater Poland (Poland) between 2006 and 2018. The second aim was to compare the present epidemiological data to records obtained in the previous years (1998–2003) in pediatric population aged 0–14 years from the same area.

## Methods

In this cohort study, data were collected for children and adolescents living in Greater Poland (west-central Poland), which is second in area and third in population among Poland’s sixteen voivodeships, with an area of 29,826 square kilometers and a population of close to 3,5 million (9,24% of the total Polish population) [12]. Pediatric diabetes care is centralized in Greater Poland, and all newly diagnosed with diabetes children are referred to the Department of Pediatric Diabetes, Clinical Auxology and Obesity in Poznan (Poznan University of Medical Sciences). **The secondary source of validation was the Diabetes Outpatient Clinics**,** which were located in different parts of Graeter**,** Poland province. The degree of completeness of the registry overall has been estimated at 95.0%.** T1D cases were obtained through **a retrospective population-based registry** [13]. **Hospital inpatient and/or diabetes outpatient clinic medical records** were reviewed and met the following criteria: new onset T1D (confirmed by the presence of diabetes-associated autoantibodies) diagnosed between 1 January 1998 and 31 December 2018, age 0–14 years, permanent residency at the time of diagnosis in the study area, which was defined geographically to correspond with administrative and census boundaries of Greater Poland and Polish origin. New cases of T1D were diagnosed in accordance with the International Society for Pediatric and Adolescent (ISPAD) Diabetes criteria [9]. To confirm autoimmune diabetes origin typical autoantibodies were tested. GAD-ab and IA2-ab were measured by the RIA test kits, GAD-ab Assay (IgG) (positivity:>1 U/ml) and IA-2-ab Assay (IgG) (positivity:>1 U/ml), respectively. [GAD and IA2 RIA. EUROIMMUN, Lubeck, Germany]. Level of IAA was determined the RIA test kits [AIA-100, DIAsource Immunoassay, S.A, Louvain-la-Neuve, Belgium]. The upper limit of the normal range was 5,5% U/mL, according to the manufacturer’s recommendation. **In the case of negative autoantibodies**,** the clinical course of diabetes was tracked in the outpatient clinic**,** and patients were clinically diagnosed as having T1D.** The study protocol was approved by the Ethics Committee at the Poznan University of Medical Sciences.

### Statistical analysis

The overall population size was taken from the Central Statistical Office (11). Total, sex-, and age-specific IRs per 100,000 person-years were calculated for each calendar year. A direct standardization method was used to estimate age and sex standardized rates, assuming a reference population comprising equal numbers in each of the sex- and age-specific groups (0–4, 5–9, and 10–14 years. The monthly time series decomposition was used to identify long-term trends, seasonal variations, and irregular fluctuations in the occurrence of the T1D among children over the 2006–2018 time frame. To model the recognized T1D data, a Poisson regression model was performed in order to check if any covariate (year, gender and age category) may influence the risk of T1D diagnosis. The result are presented as incidence rate ratio (IRR) and its 95% confidence interval (CI). All test were considered significant at *p* < 0.05. The calculations were performed by STATA 17 (StataCorp. 2021. Stata Statistical Software: Release 17. College Station, TX: StataCorp LLC).

## Results

During the 13-year period from 2006 to 2018, a total of 1,484 children < 15 years of age with newly diagnosed T1D from Greater Poland were recorded. The occurrence of T1D diagnosis was more frequent in males and it was reported in 827 boys (55.7%) and 657 girls (44.3%). The mean age at diagnosis was 9.02 ± 3.8 years. The rate of ketoacidosis at the time of T1D diagnosis was high with 36% during the study period. Characteristics of the study population at baseline are shown in Table 1.

**Table 1:** Baseline characteristic of the study population (2006-2018)

Variable	Mean ± SD
Total number of patients	1,484
Sex (F: M)	657:827
Age (year)	9.02 ± 3.8
DKA (%)	36.0
NGSP HbA1c (mmol/mol); [%]	97 ± 9 [11.4 ± 2.8]
Glycemia at admission (mmol/l); [mg/dl]	23.3 ± 10 [418 ± 180]
C-peptide (pmol/l)	0.3 ± 0.8
Diabetes related autoantibodies	
Positive GAD-ab (%); [n]	66.0 [979]
GAD-ab titer (U/ml)	20.48 ± 41
Positive IA2-ab (%); [n]	74.4 [1108]
IA2-ab titer (U/ml)	13.53 ± 15.47
Positive IAA (%); [n]	46.7 [693]
IAA titer (%)	7.73 ± 7.83

Data are expressed as mean; *HbA1c* glycated haemoglobin; *DKA* diabetic ketoacidosis

The growing trend has been observed over a 13-year period. In 2006 the IR of childhood T1D was 9.2/100,000 per year (95% CI 7.5–11) and raised up to 30.8/100,000 per year (95% CI 27.6–34) in 2018 among children aged 0–14, with the peak incidence recorded in last year of the study. The highest annual incidence rates were mostly reported among those aged 5–9 years and aged 10–14 years. However, the prevalent increase of T1D incidence was reported in all age groups, with comparable speed of incidence growth. The IR has increased between 3.3 to 3.5-fold depending on the age group (3.3-fold in 10–14 age group and 3.5-fold in 5–9 age group). Among the youngest age group, the IR was 5.8/100,000 per year (95% CI 3.3–8.4) at the beginning of the study and reached 19.9/100,000 per year (95% CI 15.4–24.4) in the last year of the study. While, in 5–9 and 10–14 age groups, the IR was registered at 9.5/100,000 per year (95% CI 6.3–12.7) and 11.8/100,000 per year (95% CI 8.5–15.1) in 2006 and rose to 34.1/100,000 per year (95% CI 28.3–39.9) and 38.4/100,000 per year (95% CI 32.1–44.7) in 2018, respectively. The IRs in the four age groups (0–4, 5–9, and 10–14 years) by sex are shown in Table 2.

**Table 2:** Incidence rates (per 100,000 person-years) of T1D in Greater Poland during 2006 to 2018, according to sex and age groups (age adjusted)

Year	Overall	Female	Male
	IR (95%CI)	IR (95%CI)	IR (95%CI)
**Age group 0 to 4**			
2006	**5.8** (3.3–8.4)	**3.6** (0.48–7.7)	**7.9** (2.0-13.8)
2007	**12.5** (8.8–16.2)	**18.8** (9.6–28.0)	**6.6** (1.3–11.9)
2008	**7.1** (5.2–11.1)	**5.6** (0.7–10.6)	**10.5** (4.0-17.1)
2009	**10.5** (7.2–13.7)	**9.8** (3.4–16.1)	**11.1** (4.5–17.7)
2010	**15.9** (12.0-19.8)	**18.5** (9.9–27.0)	**13.5** (6.4–20.5)
2011	**13.8** (10.2–17.4)	**13.2** (6.0-20.4)	**14.4** (7.1–21.6)
2012	**17.4** (13.3–21.5)	**16.4** (8.4–24.4)	**18.3** (10.1–26.6)
2013	**17.4** (13.3–21.5)	**20.0** (11.0–29.0)	**15.0** (7.4–22.5)
2014	**13.1** (9.5–16.8)	**13.0** (5.6–20.3)	**13.3** (6.1–20.5)
2015	**16.7** (12.5–20.8)	**11.0** (4.2–18.0)	**22.0** (12.5–31.3)
2016	**21.5** (16.8–26.2)	**18.9** (10.0-27.8)	**24.0** (14.2–33.8)
2017	**13.3** (9.6–17.0)	**7.6** (1.98–13.3)	**18.6** (10.0-27.2)
2018	**19.9** (15.4–24.4)	**14.0** (6.4–21.6)	**25.5** (15.5–35.5)
**Age group 5 to 9**			
2006	**9.5** (6.3–12.7)	**6.9** (1.4–12.4)	**11.9** (4.9–19.0)
2007	**21.2** (16.4–26.1)	**26.0** (15.1–36.8)	**16.7** (8.3–25.2)
2008	**22.8** (15.7–25.2)	**22.8** (12.6–33.2)	**18.2** (9.3–27.1)
2009	**24.8** (19.5–30.1)	**17.1** (8.14–26.1)	**32.1** (20.2–43.9)
2010	**25.7** (20.3–31.1)	**21.7** (11.7–31.7)	**29.5** (18.2–40.8)
2011	**20.7** (15.9–25.5)	**26.0** (15.2–37.0)	**15.6** (7.5–23.8)
2012	**25.6** (20.4–30.9)	**20.7** (11.2–30.3)	**30.2** (19.0-41.4)
2013	**27.1** (21.9–32.4)	**33.0** (21.2–44.8)	**21.7** (12.4–30.9)
2014	**27.0** (21.9–32.2)	**22.2** (12.7–31.6)	**31.6** (20.6–42.5)
2015	**21.9** (17.3–26.4)	**25.7** (15.6–35.7)	**18.3** (10.0-26.5)
2016	**25.6** (20.7–30.5)	**23.3** (13.8–32.9)	**27.7** (17.6–37.8)
2017	**25.7** (20.8–30.7)	**23.5** (13.9–33.1)	**27.8** (17.7–38.0)
2018	**34.1** (28.3–39.9)	**30.4** (19.4–41.5)	**37.6** (25.6–49.5)
**Age group 10 to 14**			
2006	**11.8** (8.5–15.1)	**13.5** (6.5–20.6)	**10.2** (4.1–16.1)
2007	**10.3** (7.2–13.4)	**7.0** (1.8–2.3)	**13.4** (6.4–20.4)
2008	**17.4** (13.7–22.1)	**18.9** (10.2–27.6)	**16.9** (8.9–25.0)
2009	**20.1** (15.6–24.7)	**16.3** (8.1–24.6)	**23.8** (14.0-33.5)
2010	**20.6** (16-25.2)	**21.2** (11.6–30.7)	**20.0** (11.0–29.0)
2011	**23.9** (18.8–28.9)	**19.4** (10.2–28.7)	**28.0** (17.3–38.8)
2012	**18.2** (13.8–22.7)	**17.6** (8.7–26.5)	**18.8** (9.9–27.8)
2013	**30.0** (23.3–34.7)	**19.1** (9.7–28.4)	**38.4** (25.5–51.3)
2014	**26.4** (20.9–31.8)	**18.1** (8.9–27.3)	**34.0** (21.9–46.4)
2015	**26.4** (20.9–31.8)	**24.1** (13.5–34.7)	**28.4** (17.3–39.6)
2016	**32.9** (26.9–39)	**26.2** (15.3–37.2)	**39.2** (26.2–52.2)
2017	**28.5** (23.0–34.0)	**26.6** (15.7–37.4)	**30.3** (19.1–41.6)
2018	**38.4** (32.1–44.7)	**29.7** (18.5–41.0)	**46.5** (32.9–60.0)
**Age group 0 to 14**			
2006	**9.2** (7.5–11.0)	**8.4** (4.9–11.9)	**10.0** (6.4–13.7)
2007	**14.4** (12.2–16.7)	**16.7** (11.8–21.6)	**12.3** (8.2–16.3)
2008	**15.6** (13.2–17.7)	**16.0** (14.1–17.2)	**15.5** (13.1–17.3)
2009	**18.2** (15.7–20.7)	**14.3** (9.7–18.8)	**21.9** (16.5–27.4)
2010	**20.5** (17.8–23.1)	**20.4** (14.9–25.7)	**20.6** (15.3–25.8)
2011	**19.2** (16.7–21.8)	**19.7** (14.0-24.5)	**19.2** (14.1–24.3)
2012	**20.3** (17.7–23.0)	**18.2** (13.0-23.6)	**22.3** (16.8–27.8)
2013	**24.3** (21.4–27.2)	**24.1** (18.2–29.9)	**24.5** (18.7–30.2)
2014	**22.1** (19.3–24.8)	**17.8** (12.7–22.8)	**26.1** (20.2–32.0)
2015	**21.5** (18.8–24.2)	**20.3** (14.9–25.7)	**22.6** (17.1–28.1)
2016	**26.5** (23.5–29.5)	**22.7** (17.1–28.4)	**30.0** (23.7–36.4)
2017	**22.5** (19.7–25.2)	**19.2** (14.0-24.4)	**25.6** (19.8–31.4)
2018	**30.8** (27.6–34.0)	**24.7** (18.9–30.6)	**36.5** (29.6–43.4)

*CI* confidence interval

Taking into account the Poisson regression analysis for available data between 2006 and 2018 it might be concluded that all analyzed factors increased the risk of T1D among children. The IRR has been increasing for each year referring to the year 2006. A higher increase has been observed from year 2013. Interestingly, a male predominance was found in the study. Male patients were more frequently diagnosed with T1D, the IRR has reached 1.24 (95% CI 1.13–1.37). In terms of age at diagnosis, a bimodal distribution of T1D occurrence was recorded. The first peak in IRR of T1D was observed in the age group between 5 and 9 years, the IRR was estimated at 1.61 (95% CI 1.41–1.85) and it was followed by a second peak in the age group between 10 and 14 years, the IRR was around 1.47 (95% CI 1.29–1.69). The results of the Poisson regression analysis are presented in Table 3.

**Table 3:** Incidence rate ratios and its 95% CI for analyzed parameters in children < 15 years of age from Greater Poland during 2006 to 2018

Parameter	IRR	95%CI	*p*-value
**Year**			
2006	1-ref.		
2007	1.58	[1.14–2.19]	NS
2008	1.65	[1.19–2.3]	NS
2009	1.99	[1.45–2.74]	< 0.001
2010	2.2	[1.61–3.01]	< 0.001
2011	2.17	[1.59–2.98]	< 0.001
2012	2.28	[1.67–3.13]	< 0.001
2013	2.82	2.08–3.82]	< 0.001
2014	2.56	[1.88–3.48]	< 0.001
2015	2.36	[1.73–3.22]	< 0.001
2016	3.19	[2.37–4.31]	< 0.001
2017	2.58	[1.9–3.51]	< 0.001
2018	3.64	[2.71–4.88]	< 0.001
**Gender**			
F	1-ref.		
M	1.24	[1.13–1.37]	< 0.001
**Age**			
0–4 years	1-ref.		
5–9 years	1.61	[1.41–1.85]	< 0.001
10–14 years	1.47	[1.29–1.69]	< 0.001

*IRR* incidence rate ratios, *CI* confidence interval, *NS* non-significant

**Fig. 1:**
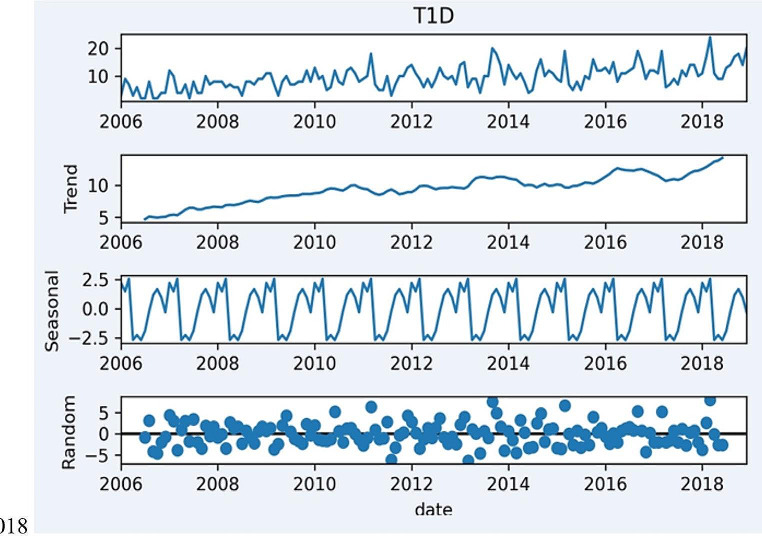
Decomposition of the T1D incidence time series in children aged 0–14 from Greater Poland between 2006–2018. Decomposition of time series – additive model. $\:{y}_{t}={T}_{t}+{S}_{t}+{R}_{t}$, where $\:{T}_{t}$ - Trent component of time series estimated with a moving averages smoothing procedure. $\:{S}_{t}$- Seasonal component of time series estimated an effect for each month of the yearas after de-trending of the original time series $\:{y}_{t}$. $\:{R}_{t}$- Random (irregular) component for the additive model determined as $\:{R}_{t}={y}_{t}-{T}_{t}-{S}_{t}$

A majority of the children were diagnosed during fall and winter. Spring and early summer was the seasons with the lowest number of new cases compared to the other seasons. Seasonal decomposition of the number of T1D diagnosed children has proven an increasing trend as well as the some seasonal component. The further analysis of seasonal component has shown that highest number of diagnosed children was observed in March. Figure 1 and Table 4 summarized seasonal trends of diagnosis of T1D in children aged 0–14 from Greater Poland.

**Table 4:** The aggregated seasonal component given for the months January–December

Month	Jan	Feb	Mar	Apr	May	Jun	Jul	Aug	Sep	Oct	Nov	Dec
**Component**	1.74	1.67	1.95	-2.47	-2.44	-2.20	-1.73	-0.08	1.23	1.48	0.74	0.11

The aggregated seasonal component is the result of decomposition (after subtract the trend and irregular components). The largest seasonal component is noted in March what is mean that in March is peak in T1D diagnosis in each year

Finally, comparison of the present epidemiological data to records obtained from 1998 to 2003 in children aged 0–14 years from the same area evidently showed dynamic changes in trends in the incidence rates of T1D in Greater Poland. Over a 20-year period, the incidence rate dramatically rose from 8.4/100,000 per year (95% CI 6.7–10.4) to 30.8/100,000 per year (95% CI 27.6–34). It indicates that Greater Poland experienced almost 3.6-fold increase in the incidence rate of childhood T1D in a relative short period. The incidence rates in children < 15 years from Greater Poland between 1998 and 2003 and 2006–2018 are presented in Table 5.

**Table 5:** 20-year trend of T1D incidence in children ≤ 14 years of age from Greater Poland

Year	IR (95%CI)
	Age group 0 to 14
1998	**8.4** (6.7-10.14)
1999	**9.6** (7.8–11.7)
2000	**7.6** (5.9–9.5)
2001	**11.8** (9.8–14.1)
2002	**12.6** (10.5–15.0)
2003	**11.2** (9.2–13.5)
2006	**9.2** (7.5–11.0)
2007	**14.4** (12.2–16.7)
2008	**15.6** (13.2–17.7)
2009	**18.2** (15.7–20.7)
2010	**20.5** (17.8–23.1)
2011	**19.2** (16.7–21.8)
2012	**20.3** (17.7–23.0)
2013	**24.3** (21.4–27.2)
2014	**22.1** (19.3–24.8)
2015	**21.5** (18.8–24.2)
2016	**26.5** (23.5–29.5)
2017	**22.5** (19.7–25.2)
2018	**30.8** (27.6–34.0)

*IR* incidence rate ratios, *CI* confidence interval

## Discussion

The IR of T1D is growing at an alarming rate worldwide, with the most rapid rate of increase contributed by countries considered earlier as those having a low incidence of T1D, such as Poland [3, 9]. The first epidemiological data from Greater Poland was published by Rewers et al., estimating an average IR in children aged 0–16 at 3.5/100,000 per year in 1970–1984 and 6.6/100,000 per year in 1982–1984 [14, 15]. In accordance with other registries, the IR of T1D in children aged 0–14 years living in Greater Poland has increased significantly over a period of 20 years, from 8.4/100,000 per year in 2008 to 30.8/100,000 per year years in 2018. Up to date, in Poland, no national registry of childhood T1D incidence has been proposed, however, the epidemiological data are constantly documented by few regions of the country. Most data on the incidence of T1D in children come from the Silesian center [10, 16–18]. The findings from this center are in line with results from the our study. In Silesia, the mean standardized IR significantly increased from 5.8/100,000 per year (1989-1994) to 18.94/100,000 per year (2007‐2012) [18]. A similar trend was observed in the multicenter study performed in eastern and central Poland. Over a period of five years, the IR of T1D in children less than 17 years rose around 1.5-fold, from 12.84/100,000 per year in 2010 to 18.46/100, 000 per year in 2014, and the increase was even higher in children < 15 years of age (21.27/100,000 per year in 2014) [19]. Similarly, high IRs of T1D in children are also reported in other European countries, including Czech Republic with 28.4/100,000 per year (2021), Germany with 24.4/100,000 per year (2000–2021) or Croatia with 17.44/100,000 per year (2004‐2012) [20–22]. However, in some high-incidence countries the overall IR of T1D reached a plateau or even decreased [23–25]. Somewhat surprisingly, a 16-year observational study conducted between 2003 and 2018 in children under 15 years of age from Finland showed the decline in overall incidence rate, which fall from 57.9/100,000 per year (2003‐2006) to 52.2/100,000 per year (2015‐2018). The results of this study were unexpected, particularly bearing in mind the previous data showing a fast rise in T1D incidence in Finnish childhood population [25].

Previous European studies suggested that the highest IR of T1D was in children aged 0–4 years [26, 27]. Nevertheless, more recent data may suggest changes in incidence rates depending on age. The study from Finland reported the most significant decrease in IR of T1D in children younger than 7 years of age, while the drop was less apparent in older children. At the same time, a rise in median age at diagnosis was also noticed, interestingly an increase was more prominent in boys only [25]. In our study, in the first years of observation (1998–2003), the highest IR was observed in 10–14 age group, Then, since 2009, the peak of the age-specific prevalence shifted to 5–9 age group. This trend remained till 2013 (except 2011), after that the IR was comparable high between those two age groups. Nonetheless, the age of presentation of childhood-onset T1D may have a bimodal distribution pattern as regional variations are recorded in peak incidence by age group. The highest IR of T1D in children aged 5–9 was observed in Northern Africa (44.78/100,000 per year), Northern Europe (37.17/100,000 per year) and Northern America (26.31/100,000 per year), whereas the lowest in Southern Asia (0.92/100,000 per year) Eastern Asia (1.93/100,000 per year) and South America (4.47/100,000 per year). Among those diagnosed within the 10–14 age category, incidence was highest in Northern Europe (41.48/100,000 per year), Northern Africa (40.92/100,000 per year) and Northern America (33.50/100,000 per year), but lowest in Southern Asia (1.99/100,000 per year), Eastern Asia (2.78/100,000 per year) and South America (3.62/100,000 per year) [28].

Although T1D is thought to emerge in childhood or adolescence, recent studies have shown that numerically more adults than children are diagnosed every year (316,000 vs. 194,000 incident cases worldwide in 2021), with a mean diagnosis age of 32 years [2]Data from the available studies suggest that T1D incidence in adults does not decline with increasing age [29, 30]. German study, which for the first time attempted to estimate the T1D epidemiology for the whole German population between 2010 and 2040, projected the relative increase of the people having T1D from 252 000 (+ 1% compared with 2010) to 327 000 (+ 32%) in 2040, with the peak of the age-specific prevalence shifting toward older ages [29]. However, data on adult-onset T1D are scarce due to several factors, including a lack of national diabetes registries that include T1D incidence across the life span or a difficulty in distinguishing diabetes types [31, 32]. In a 2021 joint consensus statement of the American Diabetes Association and the European Association for the Study of Diabetes was published with the recommendation of using islet antibodies, in conjunction with clinical features and a C-peptide test to distinguish T1D from type 2 diabetes in adults [33].

For most autoimmune diseases there is a clear sex difference in prevalence, whereby females are generally more frequently affected than male [34]. While, the overall IR of childhood T1D is comparable between sexes [10, 35, 36]. However, in some studies, T1D occurs more frequently in males, with most pronounced sex-related differences after puberty [37–40]. Populations with higher incidences of T1D overall tend to have greater male predominance. For example, some European countries reported higher IR of T1D in boys than girls, in contrast, in Asian countries where female predominance is observed [31, 37, 39–41]. In our data base, there was a significant difference of the IR between boys and girls. Similar to our study, the other authors found the same pattern of a male predominance from age 10 onwards. Interestingly, in most previous epidemiological studies in Poland did not report sex-related differences in the overall incidence of childhood T1D [17, 18].

Up to date, a dynamic changes in T1D incidence are not understood. Genetic factors alone cannot explain such a rapid increase in incidence of T1D. The cyclic pattern of incidence rates supports the dynamic process of T1D, and more likely suggests the role of different environmental or socioeconomic factors for the increasing trend of the disease. Currently, there is considerable interest in early life factors, including maternal diet, mode of delivery, infant feeding, childhood diet, microbial exposure or air pollution impact in early childhood. Still, researchers fail to identify strong environmental risk factors and the potential interplay between environmental and genetic factors. Although, environmental influence on T1D incidence should be more deeply investigated in future studies [1].

The strength of the present study is a relatively long-term period of observation, which allows to understand trends in incidence rates of childhood T1D in Greater Poland. In addition, Greater Poland is second in area and third in population among Poland’s sixteen voivodeships, then it might be representative for polish population. However, some limitations of the current study need to be acknowledged. The current administrative division of Poland was introduced by the Polish parliament in 1998, but it came into effect on 1 January 1999. Between 1975 and 1998 there had been 49 smaller voivodeships. Previously Greater Poland was known as Poznan Voivodeship and the area and population was smaller compared to present days. It may affect the IR of T1D in children and adolescent in 1998. Another issue is that during the 20-years observation there is two years gaps in T1D incidence (2004–2005) due to difficulties in obtaining data from this period. Finally, the study is its partly retrospective design.

## Conclusions

To conclude, the present study provides valuable data on T1D in children and adolescence below 15 years of age from Greater Poland. Our work has highlighted a rapid rise in incidence of T1D in Polish pediatric population, which increased around 3.6-fold over a 20-years of observation, from 8.4 per 100 000 person-years in 1998 to 30.8 per 100 000 person-years in 2018. Significant difference of standardized IR between boys and girls was established. The rate of T1D incidence growth was comparable between all age groups, while the highest IR was mostly observed in children aged 5–9 and 10–14 years.

## Abbreviations

CI: Confidence interval
DKA: Diabetic ketoacidosis
HbA1c: Glycated haemoglobin
IDF: International Diabetes Federation
IR: Incidence rate
IRR: Incidence rate ratio
ISPAD: International Society for Pediatric and Adolescent
T1D: Type 1 diabetes
